# Visual ensemble selection of deep convolutional neural networks for 3D segmentation of breast tumors on dynamic contrast enhanced MRI

**DOI:** 10.1007/s00330-022-09113-7

**Published:** 2022-09-08

**Authors:** Masoomeh Rahimpour, Marie-Judith Saint Martin, Frédérique Frouin, Pia Akl, Fanny Orlhac, Michel Koole, Caroline Malhaire

**Affiliations:** 1 Department of Imaging and Pathology, KU Leuven, Leuven, Belgium; 2 Laboratoire d’Imagerie Translationnelle en Oncologie (LITO), U1288 Inserm, Université Paris-Saclay, Centre de Recherche de l’Institut Curie, Bâtiment 101B Rue de la Chaufferie, 91400 Orsay, France; 3 Department of Radiology, Hôpital Femme Mère Enfant, Hospices civils de Lyon, Lyon, France; 4 Department of Radiology, Ensemble Hospitalier de l’Institut Curie, Paris, France

**Keywords:** Breast neoplasms, Magnetic resonance imaging, Neural networks, computer, Image processing, computer-assisted

## Abstract

**Objectives:**

To develop a visual ensemble selection of deep convolutional neural networks (CNN) for 3D segmentation of breast tumors using T1-weighted dynamic contrast-enhanced (T1-DCE) MRI.

**Methods:**

Multi-center 3D T1-DCE MRI (*n* = 141) were acquired for a cohort of patients diagnosed with locally advanced or aggressive breast cancer. Tumor lesions of 111 scans were equally divided between two radiologists and segmented for training. The additional 30 scans were segmented independently by both radiologists for testing. Three 3D U-Net models were trained using either post-contrast images or a combination of post-contrast and subtraction images fused at either the image or the feature level. Segmentation accuracy was evaluated quantitatively using the Dice similarity coefficient (DSC) and the Hausdorff distance (HD95) and scored qualitatively by a radiologist as excellent, useful, helpful, or unacceptable. Based on this score, a visual ensemble approach selecting the best segmentation among these three models was proposed.

**Results:**

The mean and standard deviation of DSC and HD95 between the two radiologists were equal to 77.8 ± 10.0% and 5.2 ± 5.9 mm. Using the visual ensemble selection, a DSC and HD95 equal to 78.1 ± 16.2% and 14.1 ± 40.8 mm was reached. The qualitative assessment was excellent (resp. excellent or useful) in 50% (resp. 77%).

**Conclusion:**

Using subtraction images in addition to post-contrast images provided complementary information for 3D segmentation of breast lesions by CNN. A visual ensemble selection allowing the radiologist to select the most optimal segmentation obtained by the three 3D U-Net models achieved comparable results to inter-radiologist agreement, yielding 77% segmented volumes considered excellent or useful.

**Key Points:**

*• Deep convolutional neural networks were developed using T1-weighted post-contrast and subtraction MRI to perform automated 3D segmentation of breast tumors.*

*• A visual ensemble selection allowing the radiologist to choose the best segmentation among the three 3D U-Net models outperformed each of the three models.*

*• The visual ensemble selection provided clinically useful segmentations in 77% of cases, potentially allowing for a valuable reduction of the manual 3D segmentation workload for the radiologist and greatly facilitating quantitative studies on non-invasive biomarker in breast MRI.*

**Supplementary Information:**

The online version contains supplementary material available at 10.1007/s00330-022-09113-7.

## Introduction

MR imaging, alongside mammography, is one of the standard imaging modalities for the detection, diagnosis, and treatment follow-up of breast cancer [1]. Dynamic contrast-enhanced MRI (DCE-MRI) is commonly used in quantitative analysis such as radiomic studies [2] to assess the malignancy of breast lesions or tumor extensions, or predict their response to neoadjuvant therapy [3]. The analysis requires a precise segmentation of the breast tumor, but a manual delineation of the lesion is time-consuming, often tedious, and prone to inter- and intra-radiologist variability [4]. It frequently constitutes a bottleneck for the quantitative analysis of larger imaging studies using breast MRI. By providing an easy access to robust 3D quantitative features extracted from tumoral lesions, an automated 3D tumor segmentation would considerably improve the identification of non-invasive biomarkers in breast MR imaging.

The recent rise of deep learning methods has brought a renewed interest to tackle organ and lesion segmentation [4]. Deep convolutional neural networks (CNNs) have established themselves as state-of-the-art methods to segment medical images in 2D [5, 6] and in 3D [7, 8]. Many public databases and segmentation challenges are available online to train and test CNN models. Although the Medical Segmentation Decathlon [9] intends to build models that could segment multiple organs using different imaging modalities, most challenges focus on specific lesions such as brain tumors with the Brain Tumor Segmentation (BraTS) Challenge [10] or liver with the Liver Tumor Segmentation (LiTS) Challenge [11] benchmarks. To the best of our knowledge, no challenge for breast tumor segmentation using DCE-MRI has been reported.

There are fewer studies using deep learning methods to segment breast tumors using DCE-MRI than using mammograms, partly due to the availability of very large mammography datasets [12]. Studies based on DCE-MRI used well-established CNN segmentation models [13–16] based on U-Net [5], DeepMedic [17], or SegNet [18] architectures or less common models [19, 20]. Several studies [14, 16, 19, 21] took advantage of all the information given by the DCE-MRI by using the different post-contrast or subtraction (post-contrast minus the pre-contrast acquisition) images. For instance, Piantadosi et al [21] used images from three different time points (pre-contrast, first and last post-contrast images). In the same way, Hirsch et al [16] built several models taking different post-contrast images as input while Zhang et al [19] fed both post-contrast and subtraction images as input to a hierarchical CNN.

Though all these studies aimed to integrate segmentation results into a clinical workflow, the practical evaluation was only based on quantitative criteria. However, a visual assessment is still necessary to detect outliers, and should be integrated in the evaluation process. The key objective of this study was therefore to define a clinically useful tool to assist radiologists in breast lesion segmentation on DCE-MRI. Three different 3D U-Nets models were considered using either the first post-contrast T1 DCE-MRI (denoted T1c) or a fusion of T1c and subtraction images (denoted SubT1), with SubT1 images defined as the difference between the first post-contrast image and the pre-contrast image. Fusion of T1c and SubT1 images was implemented at both the image and features level, resulting in three 3D U-Net models. These models were trained and the visual ensemble selection was considered where the most optimal segmentation was selected visually by a radiologist to take advantage of the complementarity of the different U-Net models and to select the best segmentation for each patient.

## Material and methods

### Image database and ground truth definition

Breast MR images (*n* = 141) were collected from a cohort of women diagnosed with locally advanced or aggressive breast cancer (see Table 1 for clinical characteristics) and undergoing neoadjuvant chemotherapy in Institut Curie between 2016 and 2020. This retrospective study was approved by our institutional review board (IRB number OBS180204), and written informed consent was waived for it. The 3D T1 fat-suppressed DCE-MRI were acquired in a multi-center setting, with the majority of scans (77%) coming from Institut Curie with three acquisition devices (see Table 2). A dedicated breast coil was used in all cases. For DCE-MRI, gadolinium-based contrast material was injected using a power injector, followed by a saline solution flush. Representative acquisition parameters for T1 fat-suppressed DCE sequences are given in Supplemental Table S1. On the whole database, in-plane voxel size varied between 0.62 × 0.62 and 1.0 × 1.0 mm, while voxel thickness ranged from 0.7 to 2.2 mm. The MRI performed outside Institut Curie were reviewed to control the quality of the images and the compliance with the recommendations of the American College of Radiology for the performance of contrast-enhanced MRI of the breast [22].

**Table 1:** Clinical information related to the 141 breast scans involved in the study. Quantitative features are given by mean values ± standard deviation; qualitative features are given by the number of cases (percentage)

Age of patients	48 ± 11 (years)
Largest diameter of tumor	29 ± 13 (mm)
Primary tumor: T stage	
I/II/III/IV	34 (24%)/83 (59%)/19 (13%)/5 (4%)
Regional lymph node: N stage	
0/I/II	77 (55%)/62 (44%)/2 (1%)
Distant metastasis: M stage	
0/I	139 (99%)/2 (1%)
Tumor type	
Ductal NOS/Others	137 (97%)/4 (3%)
Breast cancer subtype	
Luminal/HER2+/TN	41 (29%)/37 (26%)/63 (45%)

*NOS* not otherwise specified, *HER2+* human epidermal growth factor receptor 2 positive, *TN* triple negative

**Table 2:** MRI scanners and breast coils used for the training and test databases

MRI settings	Database	Cases
Institut Curie - GE Healthcare - 8 channel breast coil	Training	13
Institut Curie - Siemens Healthineers - Sentinelle breast coil	Training	50
Institut Curie - Siemens Healthineers - 18 channel breast coil	Training	16
External centers (n = 10) - GE Healthcare - breast coil	Training	21
External centers (n = 6) - Siemens Healthineers - breast coil	Training	11
**Total**	**Training**	**111**
Institut Curie - GE Healthcare - 8 channel breast coil	Test	13
Institut Curie - Siemens Healthineers - Sentinelle breast coil	Test	13
Institut Curie - Siemens Healthineers - 18 channel breast coil	Test	4
**Total**	**Test**	**30**

A set of 111 tumoral lesions was evenly segmented in 3D by two radiologists (see Supplemental Figure S1). Radiologist R1 had 15 years of experience in breast imaging while radiologist R2 had 2 years of experience. Tumors were manually segmented using the LIFEx software (v6.0, www.lifexsoft.org) [23] and were used as ground-truth labels for training and validating the CNN models. The remaining 30 lesions were segmented by both radiologists and defined as the test dataset.

### Image preprocessing

All MR images were corrected for bias field gain using the N4 algorithm as described in [24], resampled to get isotropic 1 × 1 × 1 mm voxels across the whole database then cropped in a fixed size bounding box (300 × 160 × 200 mm) ensuring that the whole breast area and armpit were included in the images. Next, images were resampled to the voxel size of 2 mm to reduce memory requirements for the segmentation model. In addition, images were normalized by dividing the intensity values of each image volume by the 95th percentile of its intensity values to avoid a normalization based on intensity outliers.

### Segmentation models

The basic architecture of the models was a 3D U-Net similar to the implementation in No New-Net [7]. The U-Net contained 4 pathways, each consisting of 2 convolutional layers with kernel size of [3] and [1, 3] (see Supplemental Figure S2 and Table S2). All convolutional layers were followed by an instance normalization and a leaky rectified linear unit (Leaky ReLU) activation function. Two fully connected layers followed by a softmax layer were added as final layers to classify the image voxels into healthy or tumoral tissue.

**Fig. 1:**
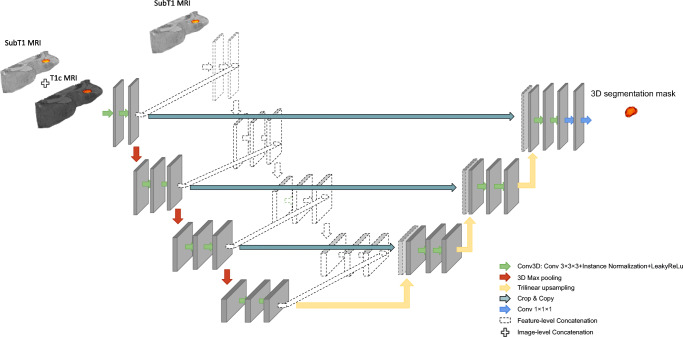
Schematic description of U-Net architecture used for image-level fusion (ILF) and feature-level fusion (FLF). The colored part represents the ILF where the T1c and SubT1 images are concatenated before being used as input for the CNN model. The dotted part is added to implement the FLF where T1c and SubT1 images are used as the input to two separate encoding parts and the extracted features from each level are concatenated

Three different configurations of the U-Net model were elaborated. The first model (referred to as “U-Net (T1c)”) was trained by the T1c image while the other two models were trained by a combination of the first post-contrast and the first subtraction images using an image- or feature-level fusion strategy to combine images. For the image-level fusion approach (denoted “U-Net ILF (T1c + SubT1)”), both MR images defined a dual-channel, used as input for the CNN model. For the feature-level fusion approach (abbreviated “U-Net FLF (T1c + SubT1)”), a U-Net architecture was used in which the encoder part consisted of two independent channels fed by the post-contrast and subtraction images, respectively. In the bottleneck of the U-Net, feature maps were concatenated and provided as the input to the decoder part, as illustrated in Fig. 1.

The models were implemented using DeepVoxNet [25], a high-level framework based on Tensorflow/Keras but specifically designed and optimized for 3D medical image data. All models were trained using a combined loss function *L* (defined by Eq. 1) defined as a weighted combination of cross-entropy (*L*_CE_) and soft Dice (*L*_SD_) losses [26]:

1 $$ L=\alpha .{L}_{\mathrm{CE}}+\left(1-\alpha \right).{L}_{\mathrm{SD}}, $$

*α* was the weighting factor of the two loss terms. For training and validation, the Adam optimizer with default Keras settings (v 2.2.4 with Tensorflow backend) was used with the initial learning rate set at 10^−3^. When the validation Dice similarity coefficient (DSC) reached a plateau, the learning rate was reduced by a factor of 5, and training was stopped when the DSC on the validation dataset did not improve during the last 500 epochs. For this implementation, a single epoch consisted of feeding 12 entire image volumes to the model with a batch size of 2. All computations were performed on the Flemish supercomputer (CentOS Linux 7) using 2 NVIDIA P100 GPUs (CUDA v11.0, GPU driver v450.57) and 1 Intel Skylake CPU (18 cores).

During training, a fivefold cross-validation was performed to determine the optimal number of epochs and a grid search was performed within a range of [0.1, 0.9] and a step size of 0.1 to find the optimal value for the hyperparameter *α*. The highest DSC was achieved when *α* was set to 0.5 to appropriately weight soft Dice and cross-entropy loss functions. At the end of the training/validation, five models were saved and then used to generate the predictions on the test dataset. For final performance comparisons, the segmentation masks were averaged over the five models of the fivefold cross-validation, and then were up-sampled to a 1 mm voxel size for comparison with the ground truth labels.

### Visual ensemble selection

Radiologist R1 visually assessed the quality of the automated segmentations obtained by the three models: U-Net (T1c), U-Net ILF (T1c + SubT1), and U-Net-FLF (T1c + SubT1) and scored the segmentation quality as “excellent,” “useful,” “helpful,” or “unacceptable.” Score 4 was given to excellent segmentations that could be used clinically without further modification. Score 3 was given to useful segmentations for which modifications (less than 25% of the total number of slices) could be achieved in a reasonable time (less than 50% of the time required for segmentation from scratch). Score 2 was given to helpful results that require substantial modifications on a larger number of slices (between 25 and 66% of the total number of slices). Score 1 was given to unacceptable results, corresponding to very large errors in the tumor delineation or cases for which tumor was not detected.

A novel patient-centric approach denoted visual ensemble selection was thus defined where, for each patient, the best segmentation was selected by Radiologist R1, when the visual scores were identical.

### Quantitative analysis

To compare segmentations, the volumes of the lesions, DSC measuring the percentage of overlap ranging from 0% (no overlap) to 100% (perfect overlap), and the 95th percentile of the symmetric Hausdorff distance (denoted HD95) measuring how far the two segmentations are distant from each other were calculated for each case of the test database. Inter-radiologist agreement was estimated by comparing the segmentations from R1 and R2. The 3D segmentations produced by the three U-Net models and the visual ensemble selection were compared to the ground truth labels defined by R1 and R2.

### Statistical analysis

Statistical analysis was performed using R software (version 4.1), with a significance level equal to 0.05. The distribution of DSC and HD95 values of segmentations obtained by the visual ensemble selection versus R1 and R2 were compared to the inter-radiologist DSC and HD95 using a Friedman test. The distribution of DSC and HD95 issued from the three 3D U-Net models were globally compared using the Kruskal-Wallis test according to the four qualitative scores and then compared using Dunn’s test and Bonferroni correction.

## Results

### Quantitative analysis

**Fig. 2:**
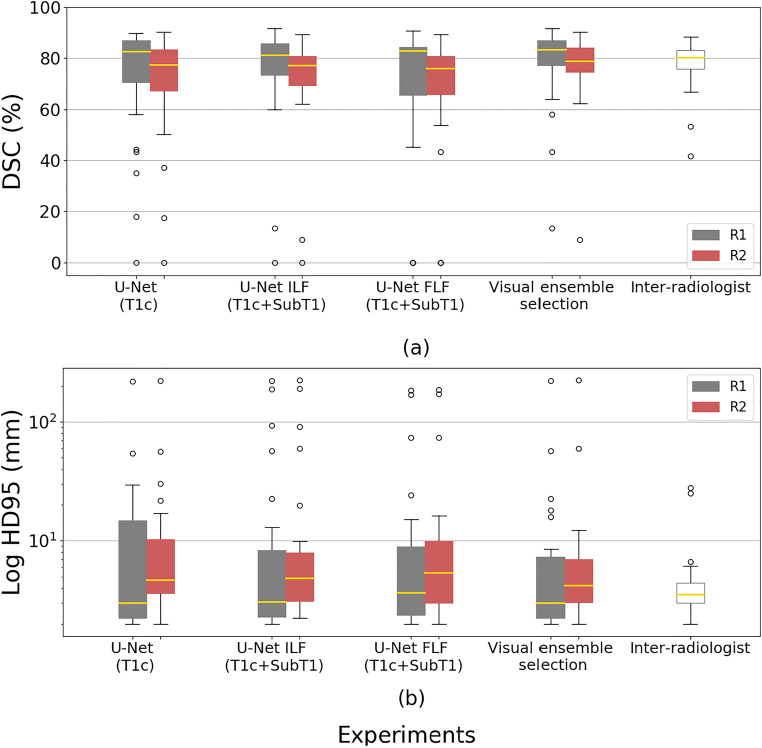
Boxplot presenting the DSC (%) (**a**) and HD95 (mm) (**b**) obtained by the different segmentation models on the test database: a U-Net using only T1c images, a U-Net trained by a combination using an image-level fusion of T1c and SubT1 images, a U-Net trained by a combination using a feature-level fusion of T1c and SubT1 images, and the visual ensemble selection. DSC and HD95 were determined using the manual delineation of the two independent radiologists (R1 and R2) as the ground truth labels. Inter-radiologist DSC and HD95 were added for comparison

Table 3 shows the volumes of the lesions as assessed by the two radiologists, the three 3D U-Net models, and the visual ensemble selection on the test database. Table 4 provides the mean and standard deviation of DSC and HD95 for the comparison of R1 and R2 segmentations (inter-radiologist criteria) and the comparison of the three 3D U-Net models and the visual ensemble selection with the segmentations provided by either R1 or R2. Figure 2 illustrates these results, providing box plots for each configuration.

**Table 3:** Volumes of lesions (mean values ± standard deviation) as estimated by the two radiologists (R1 and R2), the three CNN models, and the visual ensemble selection on the test database

Readers or models	Volumes (cm^3^)
Radiologist R1	12.9 ± 14.9
Radiologist R2	14.8 ± 17.2
U-Net (T1c)	9.8 ± 6.3
U-Net ILF (T1c + SubT1)	14.4 ± 16.0
U-Net FLF (T1c + SubT1)	11.5 ± 9.8
Visual ensemble	12.6 ± 13.5

**Table 4:** Mean values ± standard deviation of quantitative criteria (DSC and HD95) to assess the segmentation provided by three CNN models and the visual ensemble selection, using either R1 or R2 as the ground truth on the test database

	DSC (%)		HD95 (mm)	
Radiologist or model	Radiologist R1	Radiologist R2	Radiologist R1	Radiologist R2
Radiologist R2	77.8 ± 10.0		5.2 ± 5.9	
U-Net (T1c)	72.7 ± 22.8	70.6 ± 20.8	15.6 ± 40.3	15.9 ± 40.6
U-Net ILF (T1c + SubT1)	74.9 ± 20.3	71.9 ± 19.7	22.9 ± 53.2	23.6 ± 53.6
U-Net FLF (T1c + SubT1)	70.2 ± 26.1	67.3 ± 25.0	19.3 ± 45.1	19.8 ± 45.4
Visual ensemble selection	78.1 ± 16.2	76.5 ± 14.5	14.1 ± 40.8	14.1 ± 41.2

### Qualitative analysis

**Fig. 3:**
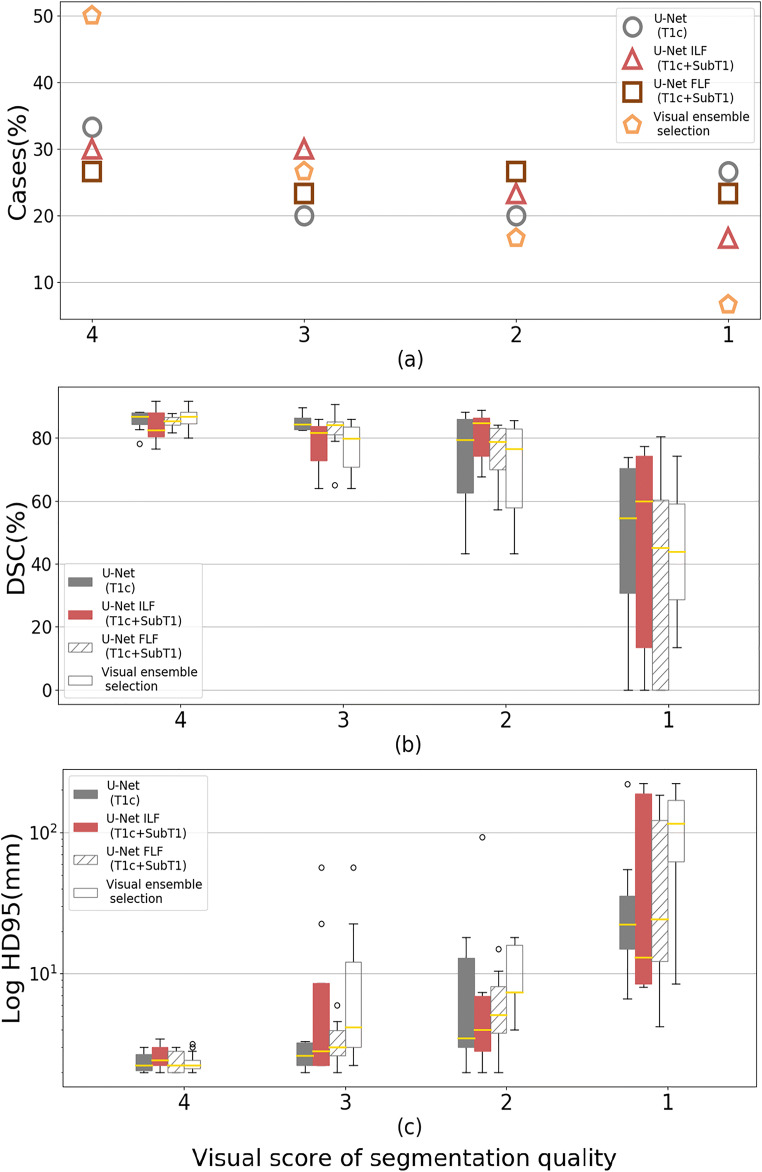
Distribution of automated segmentations according to the visual score 4 (excellent), 3 (useful), 2 (helpful), and 1 (unacceptable) (**a**) together with the boxplots presenting the DSC (%) (**b**) and HD95 (mm) (**c**). Results are shown for the different segmentation models: a U-Net using only T1c images, a U-Net using an image-level fusion of T1c and SubT1 images, a U-Net using a feature-level fusion of T1c and SubT1 images, and the visual ensemble selection

Figures 3 and S3 show the box plots obtained for DSC and HD95 according to the four quality scores of visual assessment. The three 3D U-Net models achieved comparable results with 27% to 33% of cases scored as excellent, 20% to 30% as useful, 20% to 27% as helpful, and 17% to 27% as unacceptable. When using the visual ensemble selection, 50% of cases were scored as excellent, 27% as useful, while only 23% of cases were scored as helpful or unacceptable. The global performance of the three 3D U-Net models was reduced by some outliers, while the visual ensemble selection reduced the number of outliers, which highlights the complementary role of the three 3D U-Net models.

### Statistical analysis

For the segmentations obtained by three 3D U-Net models, the mean values of quantitative criteria (DSC and HD95) on the test database were significantly different (*p* < 0.0001) for the unacceptable score (excellent, useful, helpful versus unacceptable) from the visual assessment provided by R1 (Supplemental Figure S3). Using R1 as the ground truth and based on paired rank analysis (Friedman tests), the DSC values between the segmentations provided by the visual ensemble selection and R1 were slightly better (*p* value < 0.03) than the DSC values computed from the segmentations provided by R1 and R2. There was no statistically significant difference for the HD95 results. Using R2 as the ground truth, the DSC values between the segmentation provided by the visual ensemble selection and by R2 were not significantly different from the DSC values computed using the segmentations provided by R1 and R2 (*p* value = 0.27).

### Quantitative analysis according to visual assessment

Table 5 displays the mean and standard deviation of DSC and HD95 of the visual ensemble selection compared to the segmentations provided by either R1 or R2, according to the visual assessment. For the test cases scored as excellent, the mean DSC was higher than 81%, and with the standard deviation less than 6%, showing better results compared to the inter-radiologist DSC for the whole test database. Additionally, for these cases, the mean HD95 was less than 4 mm with the standard deviation less than 2 mm.

**Table 5:** Mean values ± standard deviation of quantitative criteria (DSC and HD95) to compare the segmentation provided by the visual ensemble selection, using either R1 or R2 as the ground truth, according to the four scores of qualitative assessment on the test database

	DSC (%)		HD95 (mm)	
Visual ensemble selection	R1	R2	R1	R2
Score 4—excellent (*n* = 15)	86.3 ± 3.3	81.2 ± 6.4	2.4 ± 0.4	3.9 ± 1.5
Score 3—useful (*n* = 8)	76.9 ± 8.7	76.7 ± 8.1	13.1 ± 18.9	11.4 ± 19.7
Score 2—helpful (*n* = 5)	69.3 ± 18.1	77.4 ± 5.8	10.6 ± 6.1	8.1 ± 3.1
Score 1—unacceptable (*n* = 2)	43.9 ± 43.0	39.3 ± 42.9	116 ± 151	117 ± 154

### Illustrative cases

**Fig. 4:**
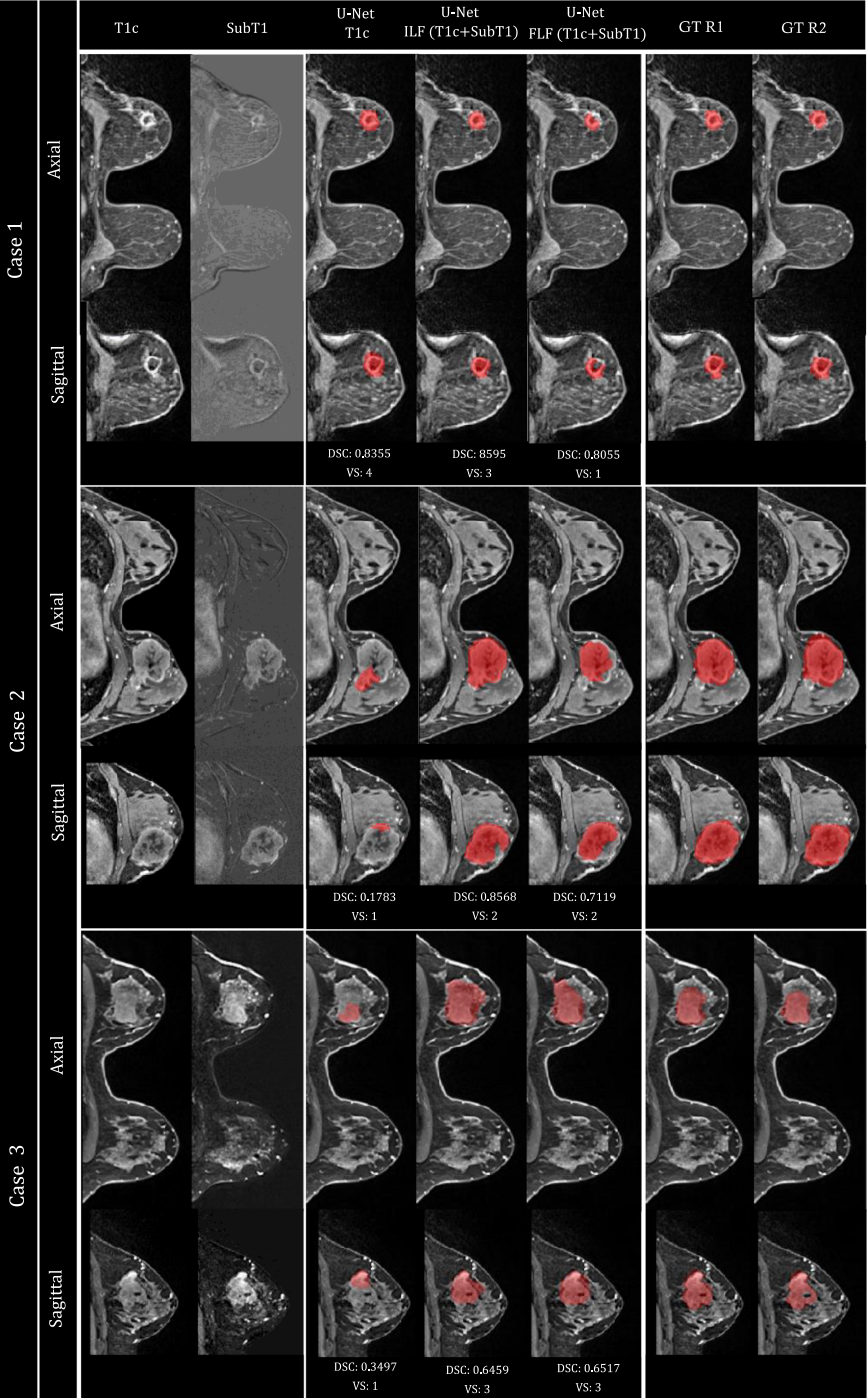
Illustration of representative segmentation results (axial and coronal views) on three cases of the test database. From left to right: T1c volume, SubT1 volume, segmentation provided by U-Net (T1c), U-Net ILF (T1c + SubT1), U-Net FLF (T1c + SubT1), ground truth (GT) provided by R1 and R2. DSC (%) and visual scoring (VS) given by R1 are included below each case. The visual ensemble selection corresponds to segmentation provided by U-Net (T1c) for case 1, and U-Net ILF (T1c + SubT1) for cases 2 and 3

Representative segmentation results of the test dataset are illustrated in Fig. 4. These exemplified cases demonstrate the interest of the visual ensemble selection while highlighting the complementary role of the three 3D U-Net models. For instance, U-Net trained with T1c images could provide excellent results (case #1) and largely underestimated volumes (cases #2 and 3). For cases #2 and #3, the 3D U-Net using image-level fusion of T1c and SubT1 images as input, provided the best segmentation, scored as helpful (13 slices out of 30 need some correction) for case #2 and as useful (7 slices out of 37 need some correction) for case #3.

## Discussion

We proposed a new CNN-based approach for breast tumor segmentation in a clinical setting. In our implementation, three 3D U-Net models were trained using different strategies: using only the post-contrast image or a combination of post-contrast and subtraction images using fusion at either the image or feature level. These three models were tested on 30 independent cases, and none of them outperformed the other two. Following a subsidiarity principle, the best segmentation among the three was ultimately selected for each patient by the radiologist, defining a visual ensemble selection. Using appropriate display tools available in LIFEx [23], the additional workload required for the visual selection is low, compared to the time that is required to check one single segmentation carefully. Furthermore, the visual ensemble selection proves to provide acceptable segmentation results in 77% of the test cases and results are globally within inter-radiologist reproducibility.

Our approach provides a 3D segmentation of breast lesions, while some of the most recent studies still segment in 2D [13, 16, 21], despite tumor volume measured by MR imaging being a strong predictor of patient survival [27]. For advanced radiomic studies or follow-up studies, 3D segmentation is also an important task to achieve [3]. The CNN models were trained using multi-centric MRI, a prerequisite for a higher generalization of these models, and they were also evaluated using a multi-scanner test dataset. Compared to many studies, for which DSC was the only evaluation criterion [15, 16], HD95 was added as a criterion for the maximal distance between two segmentations. Contrary to DSC, this criterion was not included in the loss function for the training of the models and was therefore more independent. The models designed in this study were based on the state-of-the-art U-Net architecture similar to the model proposed in [20] but without residual blocks. While Khaled et al [20] generated a breast ROI mask during the pre-processing step and used it as the input to the segmentation model along with the 3D DCE-MRI, we did not provide the U-Net models with this prior information. The prior knowledge on the breast ROI mask was also used in [16] to train the CNN segmentation models with the U-Net architecture. We only used the T1c or/and SubT1 as the input to train the U-Net models, and not a full series of DCE-MRI for training as in [20], nor T1- and T2-weighed MRI sequences as in [16]. The Deepmedic architecture with a patch-based training method was evaluated in [16] demonstrating lower performance compared to the U-Net model. This evaluation confirms our choice to use the U-Net architecture. Furthermore, performance of our U-Net models was in the same DSC range (65–80%) as reported in literature [13–16, 20, 21], though it is difficult to compare methods evaluated on different datasets with DSC computed in 2D or in 3D. The mean 3D DSC between R1 and R2 was similar to the mean 3D DSC (78–83%) for different observer combinations studied in [28]. Our database included locally advanced tumors or aggressive tumors, for which the irregular shape is difficult to segment.

The principle of an ensemble approach that combines the output of independently trained CNN models was also proposed in [20]. The authors compared a strategy of majority voting and union operation to integrate the results of several CNN models trained with different post-contrast and subtraction images. We tested the automated ensemble approaches, but they did not improve the final results (see Supplemental Table S3).

Despite the improved segmentation performance, our study had some limitations. The database used for training and testing was limited in terms of datasets, but adding progressively new cases could gradually improve the performance of the different CNN models, even if the ideal number of cases is unknown. Further use of other post-contrast images needs to be investigated as well as the potential value of adding other modalities such as diffusion-weighted images and apparent diffusion coefficient maps. Finally, the strategy we proposed is not fully automated and requires an additional visual assessment, but to the best of our knowledge, no current automated segmentation method included a self-assessment, even if a recent study [15] proposes solutions to address this issue.

This study proposes a visual ensemble selection as a new pragmatic segmentation method where the radiologist is asked to select the best segmentation among the results obtained by three different 3D U-Net models. This visual ensemble selection provided results comparable to inter-radiologist agreement with excellent or useful segmentations in 77% of the cases versus 60% of the cases for the 3D U-net model using image-level fusion of post-contrast and subtraction images, while it required little additional workload when compared to the visual evaluation of one single segmentation.

## Supplementary Information

ESM 1 (DOCX 286 kb)

## Abbreviations

CNN: Convolutional neural network
DCE: Dynamic contrast enhanced
DSC: Dice similarity coefficient
HD95: 95th percentile of Hausdorff distance
ReLU: Rectified linear unit
SubT1: Subtraction image (first post-contrast DCE-MRI minus pre-contrast DCE-MRI)
T1c: First post-contrast DCE-MRI

## Declarations

### Guarantor

The scientific guarantor of this publication is Frédérique Frouin.

### Statistics and biometry

One of the authors has significant statistical expertise.

### Methodology

• retrospective

• experimental

• performed at one institution
