# Supplementary material for: Visual ensemble selection of deep convolutional neural networks for 3D segmentation of breast tumors on dynamic contrast enhanced MRI
Source: Eur Radiol. 2022 Sep 8;33(2):959–69. doi: 10.1007/s00330-022-09113-7 (PMC9889463; doi:10.1007/s00330-022-09113-7)

**Supplementary material**

Table S1: Representative values of MR acquisition parameters for the three settings at Institut Curie

|  | GE Healthcare 8 channel breast coil | Siemens Healthineers 18 channel breast coil | Siemens Healthineers Sentinelle breast coil |
| --- | --- | --- | --- |
| TR (ms) | 6.81 | 5.2 | 5.2 |
| TE (ms) | 3.3 | 2.4 | 2.4 |
| Slice thickness (mm) | 1 | 0.9 | 0.9 |
| Spacing between slices (mm) | 1 | 0.9 | 0.9 |
| Pixel spacing (mm) | 0.82 x 0.82 | 0.91 x 0.91 | 0.91 x 0.91 |
| Bandwidth | 111 | 350 | 350 |
| Pixel bandwidth | 434 | 355 | 355 |
| Flip angle | 15 | 10 | 10 |
| Fat saturation | DIXON | SPAIR | SPAIR |
| Parallel Imaging | ARC | GRAPPA | GRAPPA |

Table S2: Parameters of U-Net model

| Pathway | Block | Number of kernels | Kernel size |
| --- | --- | --- | --- |
| 1 - EC | Conv3D | 20 | (3, 3, 1) |
| 1 - EC | Conv3D | 40 | (3, 3, 3) |
| 2 - EC | Conv3D | 40 | (3, 3, 1) |
| 2 - EC | Conv3D | 80 | (3, 3, 3) |
| 3 - EC | Conv3D | 80 | (3, 3, 1) |
| 3 - EC | Conv3D | 160 | (3, 3, 3) |
| 4 - EC | Conv3D | 160 | (3, 3, 3) |
| 4 - EC | Conv3D | 160 | (3, 3, 3) |
| 3 - DC | Conv3D | 160 | (3, 3, 3) |
| 3 - DC | Conv3D | 80 | (3, 3, 1) |
| 2 - DC | Conv3D | 80 | (3, 3, 3) |
| 2 - DC | Conv3D | 40 | (3, 3, 1) |
| 1 - DC | Conv3D | 40 | (3, 3, 3) |
| 1 - DC | Conv3D | 20 | (3, 3, 1) |
| 1 - FC | Conv3D | 20 | (1, 1, 1) |
| 2- FC | Conv3D | 1 | (1, 1, 1) |

* EC, DC, and FC stand for encoder part, decoder part and fully-connected layer.

Table S3: Mean values ± standard deviation of quantitative criteria (DSC and HD95) to assess the performance of the automated ensemble approaches: majority voting and averaging. For the averaging, the segmentation predictions obtained by the three CNN models were averaged and thresholded by 0.5.

| Automated Ensemble approach | DSC (%) | | HD95 (mm) | |
| --- | --- | --- | --- | --- |
|  | Radiologist R1 | Radiologist R2 | Radiologist R1 | Radiologist R2 |
| Majority Voting | 74.5± 21.6 | 71.6±20.5 | 20.6±50.2 | 20.6±50.5 |
| Averaging | 74.9±22.0 | 72.3±20.8 | 14.0±25.13 | 14.24±25.1 |

Figure S1: Flowchart for the definition of training and test datasets

Figure S2: Overview of the 3D U-Net model


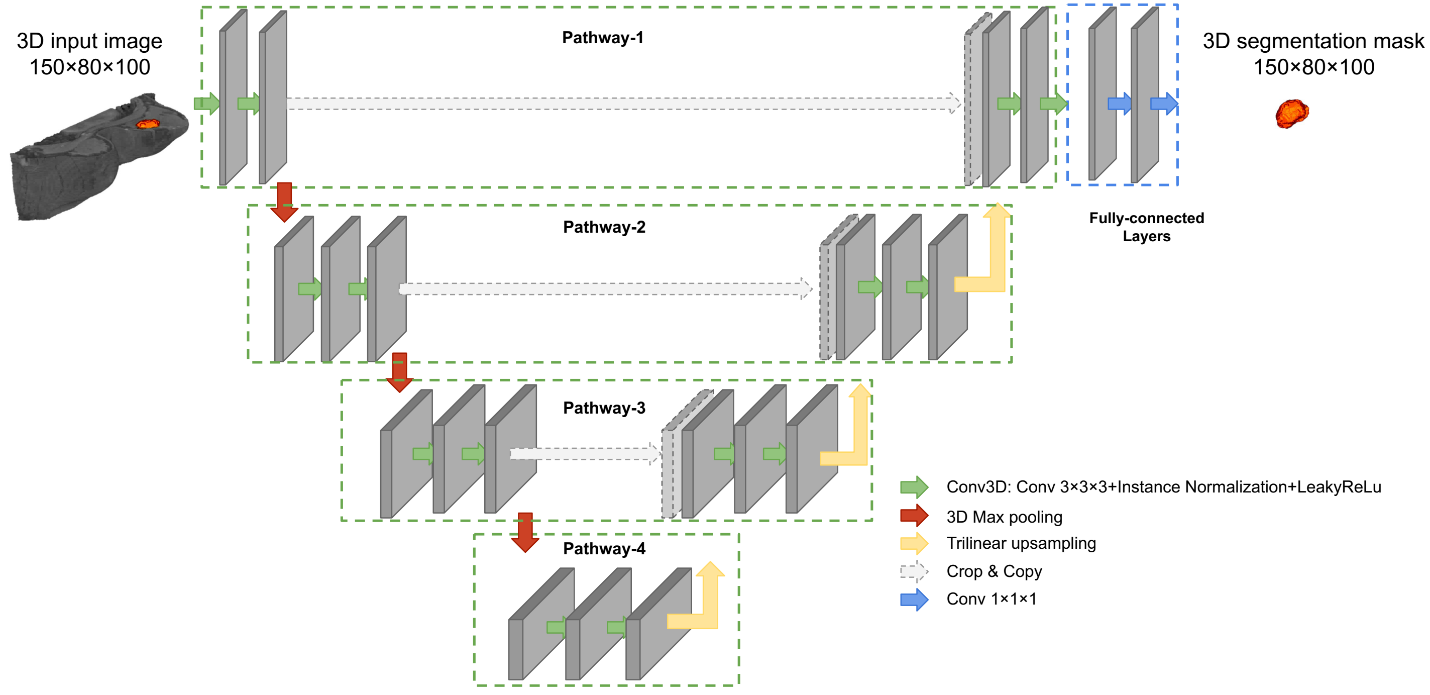


Figure S3. Statistical analysis performed on the quantitative criteria (DSC and HD95) obtained by the three 3D U-Net models and using R1 as the ground truth according to the four visual scores (excellent, useful, helpful, and unacceptable), showing some significant differences of the mean values of DSC and HD95 according to the visual scores (****p-value<0.0001, ** p-value<0.01).


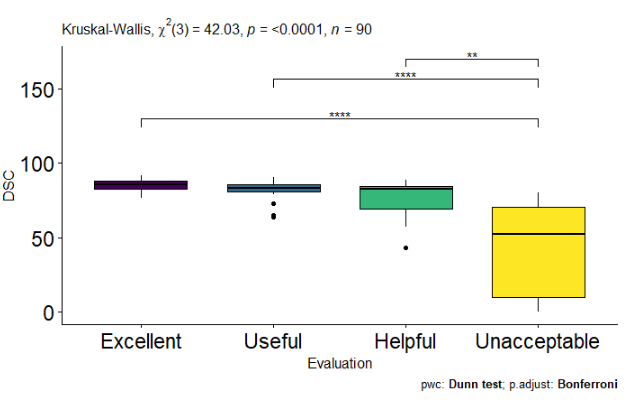


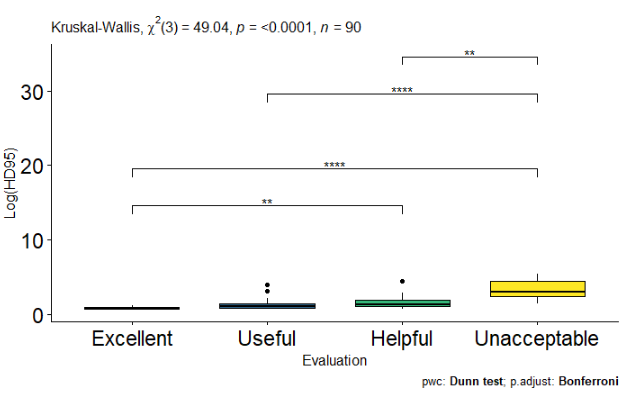

Supplement: Supplementary file 1 — (DOCX 286 kb) [file 330_2022_9113_MOESM1_ESM.docx]
